# New SARS-CoV-2 Variant from Jordan

**DOI:** 10.1128/MRA.00532-21

**Published:** 2021-07-01

**Authors:** Lo’ai Alanagreh, Mustafa Ababneh, Abdel-Ellah Al-Shudifat, Mai Ajlouny, Hanan Abu-Shaikh, Foad Alzoughool, Mohammad-Borhan Al-zghoul, Manar Atoum, Azmi Mahafzeh

**Affiliations:** a Department of Medical Laboratory Sciences, Faculty of Applied Medical Sciences, The Hashemite University, Zarqa, Jordan; b Basic Veterinary Sciences, School of Veterinary Medicine, Jordan University of Science and Technology, Irbid, Jordan; c Department of Internal and Family Medicine, Faculty of Medicine, The Hashemite University, Zarqa, Jordan; d Prince Hamza Hospital, Amman, Jordan; e Department of Pathology, Microbiology and Forensic Medicine, School of Medicine, University of Jordan, Amman, Jordan; Queens College CUNY

## Abstract

A variant of severe acute respiratory syndrome coronavirus 2 (SARS-CoV-2) from Jordan was identified during the second wave of infection. The genome of this variant has a unique set of mutations that suggest local evolution. Due to the continuous emergence of new variants worldwide, molecular surveillance is crucial for fighting the pandemic.

## ANNOUNCEMENT

Severe acute respiratory syndrome coronavirus 2 (SARS-CoV-2; the causative agent of COVID-19) belongs to the family *Coronaviridae* and the genus *Betacoronavirus* (1, 2). In viral outbreaks, new variants are expected to emerge as part of the virus’s natural evolution (3). The assumption that SARS-CoV-2 was a slowly mutating virus began to change by the end of 2020, with the emergence of novel variants such as those reported in the United Kingdom (20I/501Y.V1/B.1.1.7), South Africa (20H/501Y.V2/B.1.351), Brazil (P.1/20J/501Y.V3/B.1.1.248), and India (B.1.617). These lineages contained mutations in the spike protein that are expected to increase the infectivity and virulence of the virus (4, 5). Since new SARS-CoV-2 variants continue to emerge globally, and Jordan is amid a second wave of COVID-19 infections, we analyzed SARS-CoV-2 variants in Jordan to see if one of these known strains had emerged. Here, we report the complete genome sequence of a SARS-CoV-2 variant isolated in Jordan.

Strain SARS-CoV-2/human/JOR/AM-HU-16/2021 was recovered (nasopharyngeal swabs) from a 57-year-old female inpatient at the Prince Hamza Hospital, Amman, Jordan. The patient was identified as positive for COVID-19 by reverse transcription-PCR (RT-PCR; Zybio, China). A QIAamp viral RNA minikit (Qiagen, Germany) was used to extract viral RNA, which was then converted into cDNA and amplified using the QIAseq SARS-CoV-2 primer panel kit (Qiagen, Germany). The Qubit 4 fluorometer was used to combine, purify, and quantify the generated 400-bp amplicons for library preparation. Nextera DNA Flex libraries were prepared and sequenced on the iSeq 100 system (Illumina) with an output of 2 × 150-bp paired ends.

The raw data were analyzed using the Illumina BaseSpace pipeline. The FASTQ Toolkit v 2.2.5 was used to remove low-quality and short reads. DRAGEN COVID Lineage v 3.5.1 was used for mapping/aligning, variant calling, and consensus sequence generation of the SARS-CoV-2 genome compared to the reference genome (Wuhan-Hu-1, GenBank accession number MN908947.3). Default parameters were used for all software unless otherwise specified.

**FIG 1:**
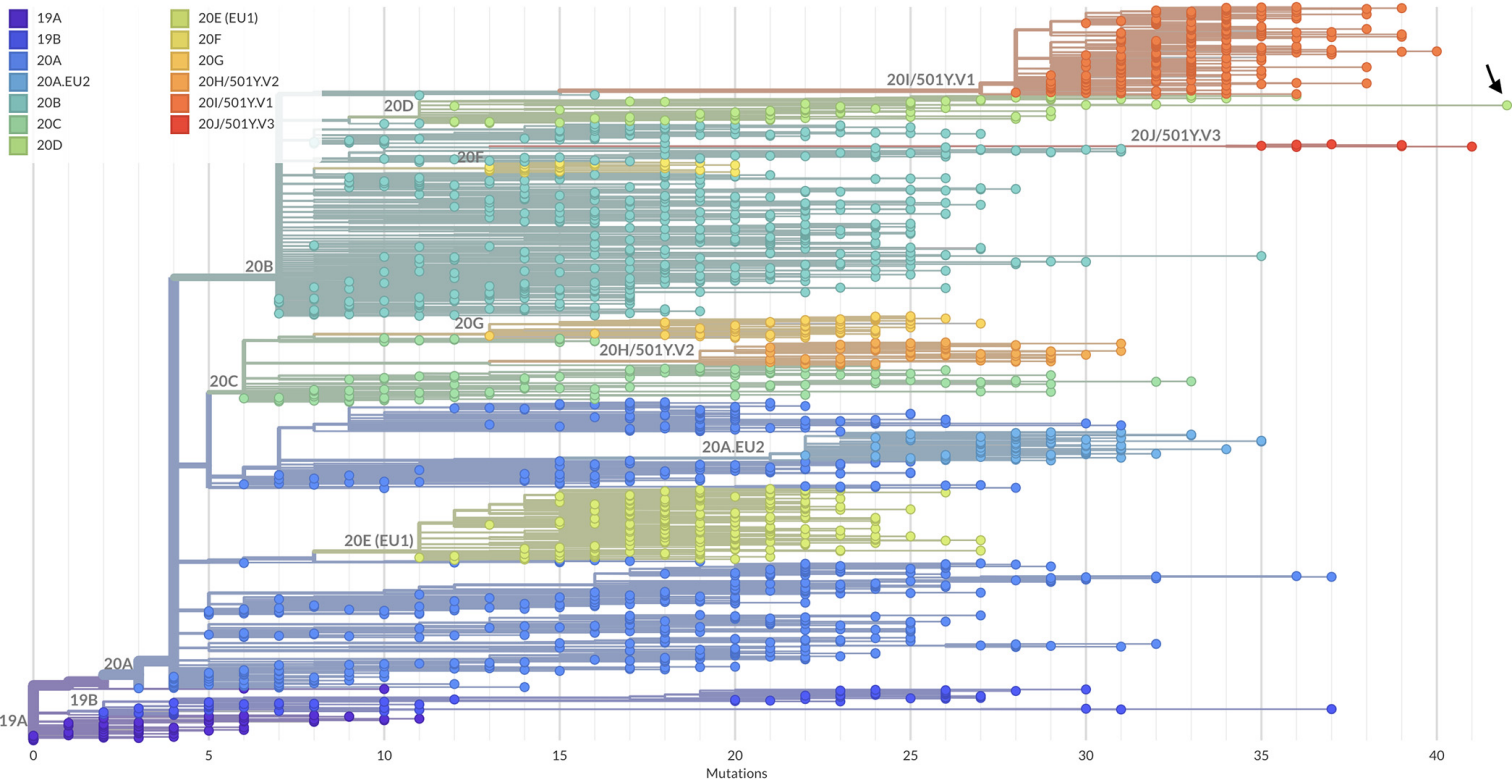
Phylogenetic relationship of SARS-CoV-2/human/JOR/AM-HU-16/2021 (black arrow) to the global SARS-CoV-2 genomes. The phylogenetic tree was constructed using the Nextclade tool v 0.14.3 (https://clades.nextstrain.org/).

Analysis allowed us to obtain a SARS-CoV-2 genome of 29,886 bp in length. From 1,157,905 reads, 1,080,169 reads were mapped, covering 99.15% of the total genome with a median coverage of 2,330×. This genome was defined by multiple spike (S) protein mutations based on viral genome sequence data (deletions 69-70, S12F, W152R, L176F, R346S, L452R, T547I, D614G, Q677H, and A899S) and is classified as a subclade of 20D (Fig. 1). The L452R mutation in the S protein is found within a known receptor binding domain that is resistant to monoclonal antibodies to the S protein (6). Clinical outcomes have yet to be determined, and the strain’s functional impact on infectivity and disease severity is unknown. Even though there are 618 genome sequences of SARS-CoV-2 from Jordan uploaded to the GISAID database, none of them is close to this variant.

### Data availability.

The genome sequence of this sample has been uploaded to the GISAID database under the accession number EPI_ISL_1336652 and to GenBank (accession number MZ266636). The raw data are available at the NCBI SRA (BioProject number PRJNA733775).
